# Three-dimensional spatial transcriptomics uncovers cell type localizations in the human rheumatoid arthritis synovium

**DOI:** 10.1038/s42003-022-03050-3

**Published:** 2022-02-11

**Authors:** Sanja Vickovic, Denis Schapiro, Konstantin Carlberg, Britta Lötstedt, Ludvig Larsson, Franziska Hildebrandt, Marina Korotkova, Aase H. Hensvold, Anca I. Catrina, Peter K. Sorger, Vivianne Malmström, Aviv Regev, Patrik L. Ståhl

**Affiliations:** 1 Klarman Cell Observatory, Broad Institute of MIT and Harvard, Cambridge, MA USA; 2 Department of Biology, Massachusetts Institute of Technology, Cambridge, MA USA; 3 Science for Life Laboratory, Department of Biochemistry and Biophysics, Stockholm University, Solna, Sweden; 4 New York Genome Center, New York, NY USA; 5 Laboratory of Systems Pharmacology, Harvard Medical School, Boston, MA USA; 6 Institute for Computational Biomedicine and Institute of Pathology, Faculty of Medicine, Heidelberg University Hospital and Heidelberg University, Heidelberg, Germany; 7 Science for Life Laboratory, Department of Gene Technology, KTH Royal Institute of Technology, Stockholm, Sweden; 8 Department of Molecular Biosciences, the Wenner Gren Institute, Stockholm University, Stockholm, Sweden; 9 Karolinska Institutet, Division of Rheumatology, Department of Medicine, Center for Molecular Medicine, Stockholm, Sweden; 10 Unit of Rheumatology, Karolinska University Hospital, Stockholm, Sweden; 11 Howard Hughes Medical Institute and Koch Institute for Integrative Cancer Research, Department of Biology, Massachusetts Institute of Technology, Cambridge, MA USA; 12 Present Address: Genentech, 1 DNA Way, South San Francisco, CA USA

**Keywords:** Gene expression, Transcriptomics

## Abstract

The inflamed rheumatic joint is a highly heterogeneous and complex tissue with dynamic recruitment and expansion of multiple cell types that interact in multifaceted ways within a localized area. Rheumatoid arthritis synovium has primarily been studied either by immunostaining or by molecular profiling after tissue homogenization. Here, we use Spatial Transcriptomics, where tissue-resident RNA is spatially labeled in situ with barcodes in a transcriptome-wide fashion, to study local tissue interactions at the site of chronic synovial inflammation. We report comprehensive spatial RNA-Seq data coupled to cell type-specific localization patterns at and around organized structures of infiltrating leukocyte cells in the synovium. Combining morphological features and high-throughput spatially resolved transcriptomics may be able to provide higher statistical power and more insights into monitoring disease severity and treatment-specific responses in seropositive and seronegative rheumatoid arthritis.

## Abstract (web summary)

Sanja Vickovic et al. use spatial transcriptomics to probe the local synovial tissue interactions in rheumatoid arthritis (RA) patients. Their results provide a valuable resource to understand the spatial organisation of cell populations in the synovium in the context of RA-associated inflammation.

## Introduction

Rheumatoid arthritis (RA) is a chronic autoimmune disease that primarily affects the joints. It consists of two broad subsets, seropositive and seronegative. Seropositive RA, comprising two-thirds of patients, who often exhibit more severe symptoms, is a classical autoimmune disease defined by the presence of rheumatoid factor (RF) or anti-citrullinated protein antibodies (ACPA)^1^. RA pathogenesis involves complex interactions between fibroblasts and cells of the innate and adaptive immune systems that lead to the imbalanced secretion of pro- and anti-inflammatory cytokines^2^. Studies of RA pathology have reported markers for an activated synovial fibroblast state^3,4^, while others revealed the contribution of adaptive immune responses in response to the production of specific cytokines^5–7^. Activation and expansion of fibroblasts in the synovial lining also contribute to changes in the extracellular matrix, further contributing to bone and cartilage destruction^8^. Current existing therapies, mainly targeting the immune cell components, can reduce symptoms and progression, but only ~60% of patients respond adequately to these treatments^9^.

Regions within sites of inflammation are filled with local accumulations of infiltrating leukocytes that form more or less organized structures. Such aggregates histologically resemble secondary lymphoid organs (SLOs) and are often termed tertiary lymphoid organs (TLOs)^10^. Patients with large and developed TLOs have been reported to respond more poorly to existing therapies^11^, but this is a topic of discussion in the field^12,13^. Recently, single-cell RNA-Seq studies have uncovered additional fibroblast and immune cell types and states associated with RA and TLOs^14,15^. However, the spatial organization of these cells and their impact on TLO pathogenesis in RA remains unknown.

We have previously developed Spatial Transcriptomics^16–18^ (ST), a method for high-throughput transcriptome profiling that retains spatial information in tissues^16^. In ST, transcriptomes are barcoded directly from frozen tissue sections. Tissue sections are placed on a glass slide covered with 1000–2000 features, each carrying a uniquely barcoded poly(d)T capture sequence enabling spatial mRNA capture. Tissue sections are then stained with Hematoxylin and Eosin (H&E) and imaged by transmitted light microscopy, followed by gentle permeabilization, mRNA capture on the poly(d)T probes, and RNA-Seq. Analysis of the resulting data provides a direct link between histology and RNA-Seq.

Here we used ST to spatially profile synovial tissues from seropositive and seronegative RA patients. To address the genomic variability and profile the TLO structures, we have studied gene expression as localized and three-dimensional (3D) views. We report the resulting gene expression signatures, spatial clusters, morphological features, and cell type composition changes at the sites of synovial inflammation. This provides a 3D, high-throughput transcriptomic view of rheumatoid arthritis-affected synovial biopsies.

## Results

### 3D spatial profiling of RA synovia

**Fig. 1:**
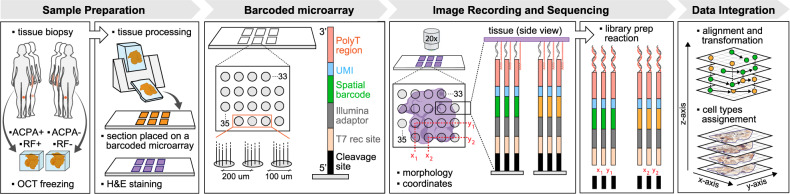
Sampling and spatial barcoding of rheumatoid arthritis samples. Synovial tissue from two patient groups, seropositive and seronegative RA, were sampled and the biopsies cryopreserved in OCT compound. The biopsies were cryosectioned and placed on a spatially barcoded microarray. Tissue sections were H&E stained and the images recorded. While recording histology, positional information of each spatial (*x*,*y*) feature was also tracked. Cells in the tissue were gently permeabilized and mRNA molecules captured on the spatially barcoded poly(d)T capture probes. The cDNA synthesis reaction was performed on the slide surface and mRNA information copied. Libraries were prepared and pair-end sequenced. The data was processed so that spatially barcoded expression information and the morphological images were registered and aligned. This resulted in spatial data transformation, interpolation, and imminent visualization.

To study the spatial organization in RA, we profiled 27 tissue sections and a total of 17,117 tissue spots by ST (“Methods”) collected from seropositive (RF^+^ACPA^+^, *n* = 3) and seronegative (RF^−^ACPA^−^, *n* = 3) RA patients at the time of joint replacement (Fig. 1, Supplementary Table 1). We optimized the technology for the tissue with the specific characteristics of synovia (“Methods,” Supplementary Fig. 1), collected profiles from consecutive sections, and aligned and interpolated the data to create a 3D view within each biopsy (“Methods,” Fig. 1). This 3D sampling approach spanned larger distances creating an exploratory multidimensional view of an RA synovial tissue biopsy.

### Distinguishing TLO structures in seropositive and seronegative RA

The biopsies from RA joints contain regions where infiltrating leukocytes (or infiltrates) organize into cell-dense areas to form TLO aggregates^19^. We detected TLOs as regions of high density and distinct cellular topology (“Methods,” Supplementary Fig. 2), where 80% of all manually-annotated infiltrates were in regions with a cell density score higher than 70% (Supplementary Fig. 3).

We then looked for spatial gene expression differences between and within infiltrates. In the first seropositive biopsy (RA1, “Methods”), analysis of spatially variable gene expression patterns revealed two clusters of infiltrate features in the TLO aggregates (“Methods,” Supplementary Fig. 4, and Supplementary Data 1) varying in the expression of multiple genes including *CD52*, *MS4A1*, and *FN1* (Supplementary Fig. 5).

**Fig. 2:**
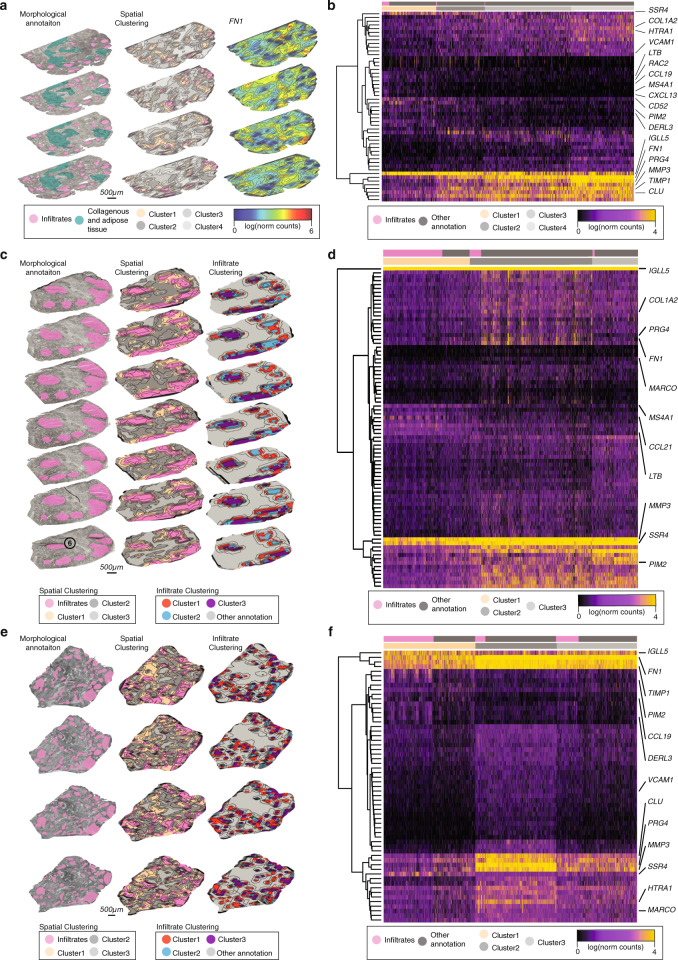
Spatial data clustering in seropositive RA. **a** Morphological annotation, spatial clustering (color code), and *FN1* spatial expression (color scale) in RA1 patient tissue volume. Color-scale denotes normalized gene expression. **b** Heatmap of RA1 gene expression (color scale) where each column represents one spatial feature and each row a gene. Spatial features (columns) have been color-coded into two morphological categories (pink; annotated infiltrates and dark gray; other annotation) and based on their spatial cluster identities as determined in **a**. Example genes (rows) have been highlighted in the image. **c** Morphological annotation, spatial clustering (color code) and infiltrate clustering (color code) in RA2 patient tissue volume. Location of Infiltrate6 (6) is highlighted in the first section in the RA2 3D volume. **d** Same as in **b** denoted for RA2 patient tissue volume. **e** Same as in **c** for RA3 patient tissue volume. **f** Same as in **b** denoted for the RA3 patient tissue volume. Scale bars represent 500 µm and are shared between the sections within the individual patient tissue volume.

Next, this spatial organization generalized by 3D ST profiling of consecutive sections in all joint-affected seropositive RA patient biopsies. Using unsupervised clustering of the regions in the entire RA1 biopsy, we identified four major spatial domains characterized by distinct spatial gene expression patterns: Cluster 1 included 87% of all annotated RA1 infiltrate data points. Clusters 2–4 included the remainder and followed radial spatial patterns at consecutively increasing distances from the infiltrate Cluster 1 regions and had lower cell density scores (Fig. 2a, b, Supplementary Fig. 6a, and Supplementary Data 2). In another seropositive biopsy (RA2), with large infiltrates that spanned most of the sampled area, unsupervised clustering partitioned the regions to three major clusters having distinct spatial expression patterns (Fig. 2c, d and Supplementary Data 2). Again, Cluster 1 corresponded to the infiltrate areas, comprising 90% of regions annotated by cellular morphology and high cell density, and the two other clusters formed a radial pattern. Key genes followed similar patterns to those in the RA1 samples, and included induction of *CD52* and *MS4A1* infiltrates (Cluster 1, *t* test, Benjamini–Hochberg (BH) adjusted, *p* ≤ 0.05) and increased *MMP3*, *FN1*, *TYROBP*, and *PRG4* expression in the surrounding areas (Clusters 2–3, *t* test, BH adjusted, *p* ≤ 0.05, Supplementary Fig. 6b). These infiltrate-specific and region-specific patterns are further generalized in RA3, a specimen from a patient having clinical characteristics similar to those of RA1–2 (Fig. 2e, f, Supplementary Fig. 6c, and Supplementary Data 2).

**Fig. 3:**
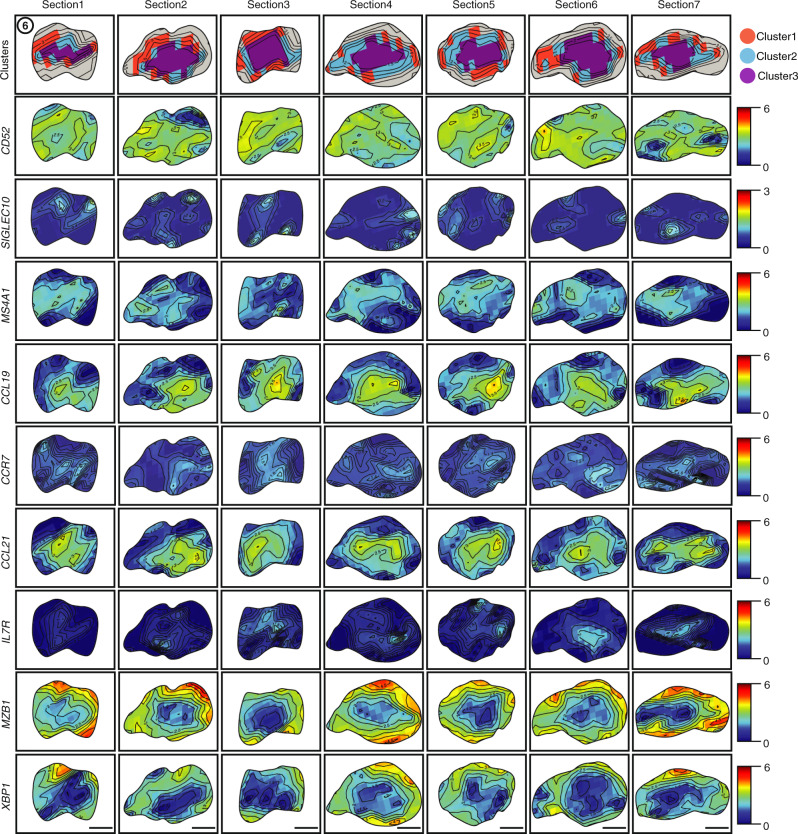
RA2 infiltrate dynamics. Zoomed in expression (color scale) of spatial clusters (color code) followed by nine example genes (rows) in the Infiltrate6 region across in RA2 sections (columns). Scale bar represents 500 µm and is listed for each individual section at the bottom of *XBP1* gene panel.

Additionally, closer examination of intra-infiltrate spatial patterns distinguished T and B cell-specific variation within the seropositive TLOs. Lymph node/TLO-associated genes (*LTB*, *CCL19* and *CCL21*) and genes associated with B cells, T cells and their cross-talk (*CXCL13*, *CD52* and *MS4A1*), were upregulated (*t* test, BH adjusted, *p* ≤ 0.05). To further explore this, we focused our analysis on intra-infiltrate changes following Infiltrate6 in RA2 (Fig. 2c, left) to identify TLO-specific spatial regions with distinct co-localization patterns (Fig. 3). We observed co-expression of *CD52* and *MS4A1* in highly localized patterns within TLO aggregates while the *CD52*/*SIGLEC10* receptor–ligand pair (“Methods”) was contained to distinct spatial clusters within the TLO sites. Upregulation of *CCL21* and *CCL19* (present in 75% of all Cluster 1 features), was also accompanied with high expression of *IL7R* in 39% of spatial measurements (Fig. 3), including matching receptor–ligand interactions with *CCR7*. While these *CCL19*^High^*CCL21*^High^ sites were restricted to the centers, *MZB1*^+^*XBP1*^+^ sites were spatially overlaid with the outer rim of the TLO-like structures (Fig. 3). These spatial gene expression patterns indicated the localized prevalence of different cell types in the TLOs.

Finally, the TLO marker genes (*CD52* and *MS4A1*) were significantly higher (*t* test, BH adjusted, p ≤ 0.05) in the same cluster denoting the TLO infiltrate structures in seronegative patient biopsies (RA4-6) (Supplementary Fig. 7, “Methods”) although the overall expression of these markers was significantly lower (*t* test, BH adjusted, *p* ≤ 0.05) in the seronegative than in seropositive patients. In seronegative patients, we did not detect similar gene expression patterns (*FN1*, *MMP3*, and *PRG4*) in the areas surrounding the TLO structures. Instead, these genes were found to be upregulated (*t* test, BH adjusted, *p* ≤ 0.05) in the infiltrates themselves. Gene Ontology (GO) analysis (“Methods”) corroborated these findings further—all seropositive patients were dominated by signatures of leukocyte migration, cell chemotaxis, and humoral immune responses; while the seronegative patients reported strong signals of extracellular matrix disassembly and regulation of cell growth.

### Substantial variation in cell composition and spatial organization in the RA synovium

**Fig. 4:**
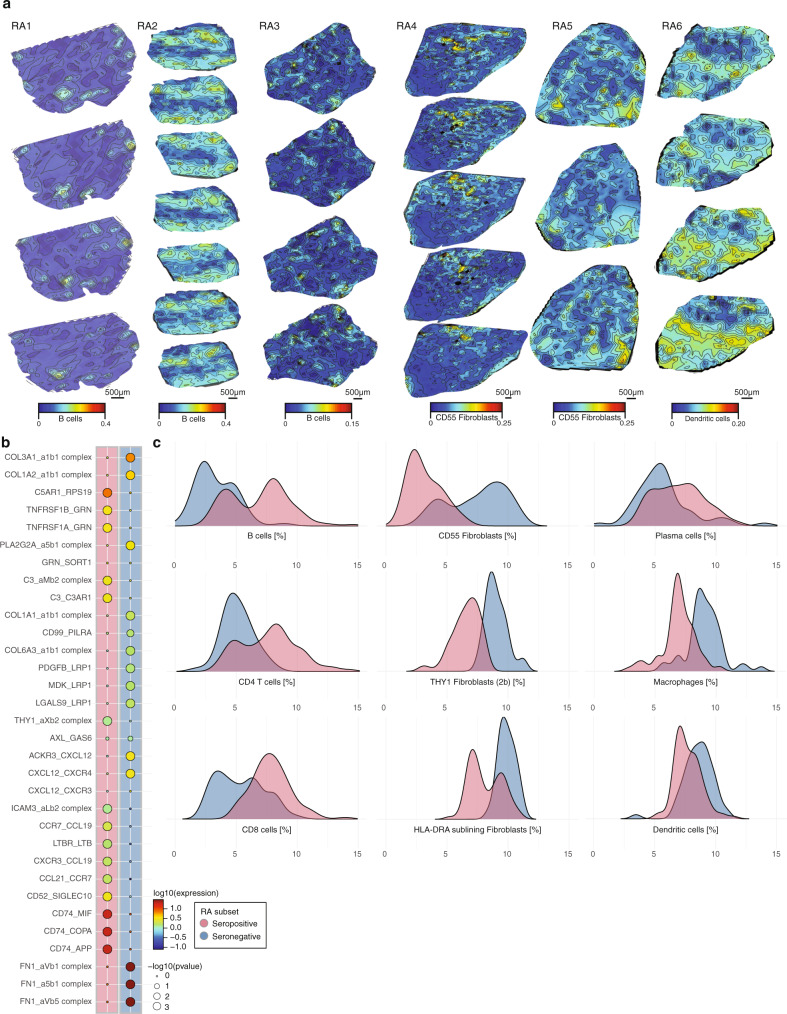
Spatial distribution of cell types in the rheumatoid arthritis synovium. **a** Abundant cell types (color scale) shown in each of the six RA patient samples (columns) and across all spatially profiled tissue sections (rows). Scale bars represent 500 µm and are shared between the sections within the individual patient tissue volume. **b** Overview of ligand–receptor interactions in RA patient subset TLOs. *p* values are indicated by circle size. Color scale indicates the means of the average expression level of interacting molecule 1 in and interacting molecule 2 in seropositive and seronegative TLOs, respectively. **c** Distributions (*y* axis) of cell-type proportions (*x* axis) for most abundant cell types in seropositive and seronegative TLOs, respectively. RA subsets color code (pink; blue) is shared between **b** and **c**.

To further explore the spatial gene expression patterns and relate these changes to the cellular composition of RA regions, we used previously published scRNA-Seq references^14,15^ to define cell-type-specific signatures, and scored our spatial regions in each tissue volume (Fig. 4a, “Methods”). Out of the 16 cell types available in the references^14,15^, plasma cells, macrophages, CD55^+^ fibroblasts, THY1^+^ fibroblasts, and specifically HLA-DRA^High^ sublining fibroblasts were found in every analyzed sample and section while spatial clusters in both patient subsets were dominated by mixtures of distinct cell types (Supplementary Fig. 8 and Supplementary Data 3).

In seropositive RA biopsies, macrophage-enriched cell areas were on average significantly co-localized with higher presence of CD55^+^ lining as well as HLA-DRA^High^ and CD34^+^ sublining fibroblast cells in the whole tissue volume (average Pearson’s *R* 0.76, *p* < 0.05, Supplementary Fig. 9a). Additionally, in specific structures spanning both TLOs and surrounding areas in RA2, macrophage areas and THY^+^ fibroblast areas co-occurred with plasma cell areas (Supplementary Fig. 9b), a trend not observed in any other seropositive sample in this study. Interestingly, while the TLO structures in seropositive RA samples were dominated by both B cells and CD4^+^ T cells, RA2 was again specific with significantly higher (*t* test, BH adjusted, *p* ≤ 0.05) abundances of CD8^+^ T cells and Tph cells. Mapping receptor–ligand interactions in the TLOs (“Methods”) confirmed that the TLO dynamics in seropositive samples was supported by complex chemokine receptor-ligand interactions between the abundant immune cells present (“Methods,” Fig. 3), while in seronegative samples; fibronectin, collagen and integrin complexes on different fibroblasts dominated the TLO sites (Fig. 4b, c). While fibroblasts in seropositive patients found in areas surrounding TLOs were associated with GO processes involved in extracellular matrix reorganization, cell metabolic processes, chondroitin sulfate synthesis, and vasculature development; fibroblasts in seronegative patients, apart from being involved in extracellular matrix reorganization, were also contributing to aminoglycan processing and antigen presentation.

In seronegative RA biopsies, no significant levels (*t* test, BH adjusted, *p* ≤ 0.05) of either B or T cell scores were seen in the tissue volumes. Conversely, DCs were substantially increased in the infiltrate areas of seronegative RA samples and their expression was not spatially correlated to plasma cell presence. This is the opposite of what was observed in all seropositive patient samples. In RA1, tissue recruitment of DCs in areas surrounding the infiltrates was associated with a decrease in plasma cells (Pearson’s *R* −0.96, *p* < 0.05). Similar was seen in RA2, a tissue volume which had the largest TLO-like structures and most B cells; DCs were few, their abundance significantly lower (*t* test, BH adjusted, *p* ≤ 0.05) as compared to all other tissue volumes and these DCs were also spatially contained to plasma cell sparse zones (Pearson’s *R* −0.89, *p* < 0.05, Supplementary Fig. 9c). The dendritic cells in seronegative patient tissue volumes were involved in antigen receptor activation, activation of immune and T cell signaling, while in seropositive patient volumes, these cells contributed mostly to apoptotic signaling.

### Connecting H&E and spatial transcriptomics reveals unified spatial clusters of morphological and molecular features

**Fig. 5:**
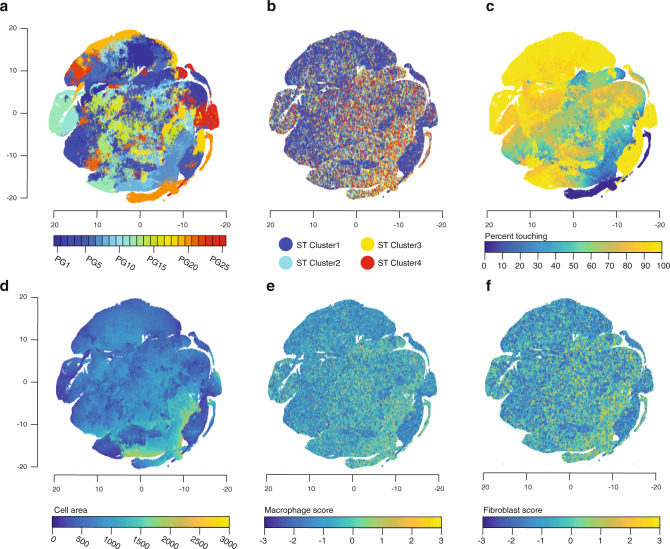
Dimensionality reduced RA2 topological features correlate with cell-type expression. **a** 25 phenotypic groups (PG) clustering visualized in tSNE projection followed by visualizations of **b** spatial clusters, **c** cell density, **d** cell area, **e** macrophage cell-type scores, and **f** THY1^+^ fibroblast cell-type scores.

Connecting morphological data to tissue-specific molecular profiles^20–23^ helps translate clinically relevant H&E information^24,25^ to spatially resolved molecular signatures. We hypothesized that distinct cellular morphological features would also be reflected in different ST profiles and in other spatial features, such as cell density. To explore this, after cell segmentation of the H&E image accompanying spatial transcriptomics, we clustered the segmented cells by their morphological features (“Methods,” Fig. 5a) and then examined their relation to other features from the H&E image and from ST.

Cluster1 (RA2), which represents the areas of infiltration, was enriched in specific H&E-derived cell clusters (Fig. 5b), and those were, as expected, also regions of high cellular density of small cells across all samples (Fig. 5c, d). Conversely, Cluster4 (in RA2) was prevalent in other H&E-defined cell clusters and those were associated with phenotypically large cell sizes (Fig. 5d), in line with abundances of larger cell types like macrophages and fibroblasts in those distinct areas (Fig. 5e, f). Across all sections, we distinguished quantitative descriptions of cellular morphology and architecture in TLO areas and related them to single-cell signatures (viewable in the histoCAT^20^ extension, “Methods”).

## Discussion

Spatially resolved genomic analysis of disease tissue holds promise for better precision phenotyping of patients and assessment of treatment responses in a manner that combines established histopathology with comprehensive molecular profiling. Here, we created an exploratory 3D spatial gene expression catalog comprising high-resolution transcriptome-wide volumetric maps correlated to morphological features. This serves, to the best of our knowledge, as the first combined morphological, spatial, and transcriptional blueprint of tissue from autoimmune disease patients, and spans multiple sections from two clinically relevant RA subsets.

The spatial clusters observed in synovial biopsies were distributed radially around the infiltrate sub-regions, further confirming the uniqueness of signals and cell types present in those areas, and highlighting the potential role of complex center-based TLO interactions in these biopsies. GO term analysis (“Methods”) revealed the spatial cell type organization throughout the 3D TLO volume transcriptionally connected to genes related to leukocyte migration, cell chemotaxis, and humoral immune responses in seropositive tissues, while the seronegative patients exhibited signals of oxidative stress and receptor-mediated endocytosis.

The synovium provides a niche cytokine-rich fibroblast-dependent environment for maintaining pro-inflammatory B cells^26^. These cell-type complexes in TLOs build specific signaling pathways through their interactions in close proximity. Mapping receptor-ligand interactions to dissect cell–cell communication (“Methods”) enabled us to systematically decode some of the cell-type responses. In our study, we report that in all seropositive patients, *CXCL13* overexpression was significant in the TLOs (*t* test, BH adjusted, *p* ≤ 0.05) and it has previously been shown overexpression of CXCL13 stimulates recruitment of B cells to the TLO sites resulting in an additional increase in localized autoantibody and cytokine production^27^. Next, we report that 34% of these *CXCL13* regions in seropositive tissue volumes co-localized with *RASGRP2* overexpression while 47% of regions exhibiting *CXCL13* downregulation were enriched for *TYROBP*. RASGRP2 had previously been implicated in arthritis development in murine models^28^ and TYROBP, also known as DAP12, when in presence of TREM2 signaling, further antagonizes cytokine production^29^. CXCL12/CCL19 expression, on the other hand, affects the spatial distributions of not only B cells, but also DCs and plasma cells in TLOs^30^. Signaling driven by these cytokines has been previously associated with overexpression of LTA and LTB, a finding recapitulated in our spatially resolved data. Again, *CXCL12*/*CCL19* expression in TLOs was significant in seropositive patients while found to be downregulated in seronegative samples (*t* test, BH adjusted, *p* ≤ 0.05). Finally, in our data, *CD74* interacts with *MIF*, *COPA*, and *APP* on surfaces of macrophages and dendritic cells in seropositive TLOs. Upon binding, MIF activates the CD74/CD44 complex and inhibiting this pathway has been suggested to have pharmacologic potential in preventing further joint destruction in RA^31^.

Finally, fibroblast cells surrounding TLOs have been associated with the propagation of TLOs and are considered marker features of lymphoid neogenesis^32^. The CD55^+^ fibroblast population was present in the synovial lining (*i.e*. outer rim of the tissue) while THY1^+^ fibroblast populations were located closer to the TLO regions in all seropositive samples. Some key genes present in these areas were *MMP3*, *FN1,* and *PRG4*. Significantly increased levels (*t* test, BH adjusted, *p* ≤ 0.05) of *MMP3* denoted areas high in extracellular matrix degradation while increasing the levels of *FN1* indicated areas high in transforming growth factor-beta (*TGF beta*) secretion. TGF beta and MMP3 are both targets already used in the clinic for monitoring RA progression as current or potential therapies due to their known contribution to joint and cartilage destruction^33,34^ whereas proteoglycans such as PRG4, secreted from the synovial fibroblasts, are involved in cartilage lubrication providing an anti-inflammatory effect^35^.

Seronegative tissue volumes lacked robust signals of ongoing adaptive immune responses and were characterized by the increased presence of DCs. DCs are involved in recruiting pro-inflammatory immune cells including macrophages, neutrophils, and monocytes in RA^36^. Specifically, in the seronegative tissue volumes, we report that CD55^+^ and THY^+^ fibroblasts, including HLA-DRA^High^ populations, as well as macrophages, were significantly overexpressed (*t* test, BH adjusted, *p* ≤ 0.05) in the TLO structures; implicating a completely different immunological drive in the sites of inflammation as compared to spatially deconvolved disease responses in seropositive tissues. The contribution of these cell-specific signatures was further confirmed by mapping receptor-ligand interactions in seronegative TLOs where FN1 binding different integrins (*e.g*. aVb1) and midkine (MDK) binding to the low-density lipoprotein receptor 1 (LRP1) promoted a proliferative environment for further production of matrix-degrading enzymes. Interestingly, the seropositive RA2 tissue with most developed TLO-like structures, apart from significantly higher abundances of B cells in the tissue (*t* test, BH adjusted, *p* ≤ 0.05), exhibited a high prevalence of CD34^+^ sublining fibroblasts and had the lowest abundances of DCs (*t* test, BH adjusted, *p* ≤ 0.05).

Combining morphological features and high-throughput spatial signatures could aid in clinical diagnosis and overall disease management of RA. Although there have been advances in obtaining multiplex protein measurements in situ^37–42^, these rely on using predefined sets of cell type markers in cyclic immunostaining, in situ sequencing barcoding schemes, and use of expensive machinery unavailable at broad scales. ST technology is compatible with conventional histological staining, has fast turnaround times, and a user-friendly laboratory set-up. While mining ST data is currently limited to deconvolution algorithms using reference scRNA-Seq data, future clinical studies using high-definition spatially resolved transcriptomics^43^ may be able to provide higher statistical power and more insights into monitoring disease severity and treatment-specific responses in seropositive and seronegative rheumatoid arthritis.

## Methods

### Patient information and sample collection

Synovial tissue biopsies from knee or hip joints were obtained during orthopedic replacement surgery. Additional patient information can be found in Supplementary Table 1. Ethical approvals were granted by the Ethics Committee of Karolinska University Hospital (2009/1262-31/3) and patients gave their informed written consent to participate in the study. The biopsies were snap frozen in isopentane prechilled with liquid nitrogen within 15 min of collection and kept at −80 °C until embedding in OCT (Sakura, The Netherlands) and sectioning could be performed.

### Spatial Transcriptomics reactions

Tissues were cryosectioned at 7 µm thickness. Each section was carefully handled inside a cryotome (CryoStar NX70, Thermo Fisher Scientific, Life Technologies, Paisley, UK) and placed onto an individual array without any direct contact between the array surface and the cryotome to avoid contamination. All sections were placed in the same fashion onto individual arrays. RA1 sections were sectioned at 21 µm distance from each other while the RA2–6 sections were consecutives. The whole slide was then warmed for 1 min at 37 °C and immediately fixed for 10 min at room temperature (RT) in a 2% formaldehyde solution (1:20 37% formaldehyde acquired from Sigma-Aldrich, Missouri, USA in 1× PBS pH 7.4). The sections were dried with isopropanol and stained with hematoxylin and eosin (H&E). To ensure proper staining, the dried sections were incubated for 7 min with hematoxylin (Mayer’s solution, Sigma-Aldrich, MO, USA) followed by 2 min in bluing buffer (DAKO, Agilent, California, USA) and 10 s in eosin Y (1:20 in slightly acidic pH 6 Tris). To record both morphological and positional information, each tissue area was imaged at ×20 resolution (Olympus, Japan) individually with a Metafer system (MetaSystems, Germany). Image stitching was performed using VSide software provided by MetaSystems. The Spatial Transcriptomics protocol was carried out^17,45,46^. Briefly, after imaging, individual sections were mildly digested for 20 min with 14 U of collagenase I in Hank’s balanced salt solution (Life Technologies, Paisley, UK) followed by a gentle wash with 0.1× saline-sodium citrate buffer (SSC) and 8 min incubation with pepsin purified from porcine gastric mucosa diluted in 0.1 M HCl. Both reactions were performed at 37 °C while the slide was kept in an ArrayIT (CA, USA) hybridization cassette to ensure reaction separation between the sections. This was followed by another gentle wash and then cDNA synthesis^17,45,46^. All reactions performed on the array surface after the digestion steps have been supplemented with 0.2× bovine serum albumin. After cDNA synthesis and tissue removal (1 h 25% beta-mercaptoethanol diluted in RNeasy lysis buffer followed by 1 h 10% proteinase K diluted in proteinase K buffer; both at 56 °C), cDNA was released from the array surface for 3 h^17,45,46^. This material served in the following library preparation steps. Following first-strand cDNA synthesis, second strand was made by nicking the RNA templates and then extending and copying the cDNA strand using DNA polymerase I^17,45,46^. The reaction was terminated with EDTA and this as well as all the following reactions were performed using an optimized library preparation protocol on a Magnatrix 8000 + (Nordiag, Sweden) pipetting station^17,45,46^. DNA hybrids were end repaired using T4 DNA polymerase and in vitro transcription reaction was performed to amplify the repaired fragments and was supplemented with an RNase inhibitor^17,45,46^. The amplified RNAs were then transcribed into cDNAs again after an Illumina adapter being directionally ligated to the 3’ ends of the amplified RNA molecules^17,45,46^. cDNAs were indexed for TruSeq LT Illumina sequencing using KAPA HotStart Hifi Ready-Mix (Roche, Switzerland)^17,45,46^. The short fragments and dimers in the finished libraries were removed^17,45,46^ and the libraries diluted to 4 pM for pair-end sequencing on either the Nextseq500 Illumina (RA1–2),  NovaSeq 6000 (RA3–5) and HiSeq 2000 (RA6) instruments.

### Labeling spatial gene activity

Sectioning, staining, imaging, and tissue digestion steps were repeated as described in the “Spatial Transcriptomics reactions” section. Next, cDNA synthesis was performed by supplementing the reaction with 25 µm Cy3-dCTPs^17,45,46^. Tissue sections were completely digested on the array surface following cDNA synthesis and the surface was then imaged with an Agilent (California, USA) G2505C microarray scanner system at 532 nm wavelength. The H&E image and Cy3-image, the latter marking spatial gene activity, were overlaid to inspect the signal-to-noise ratio under and outside the tissue section area.

### Statistics and reproducibility

#### Study design

To ensure the use of an appropriate study design, we performed power analysis^44^ to inform sample collection. First, we modeled our spatial data taking into consideration (i) the number of patients per study group; (ii) the number of replicate tissue sections per patient, and (iii) the number of replicate spatially annotated niches per each profiled spatial transcriptomics section. Using these covariates, we built a linear mixed model to simulate power analysis estimates using accurate Monte Carlo sampling needed to reach at least 80% saturation; a cut-off that is standard in the power field and assures that the effects listed in our study are real. Using a generalized mixed model for our data allowed us to account for the analysis of continuous counts and spatial non-independence. The power analysis suggested that sampling at least 3 patients in 4 replicate sections and at least 80 spatial spots per condition, would provide sufficient power to the study.

#### Data mapping, annotation, and filtering

Data were pre-processed using a recently published pipeline (v0.8.5)^47^. Raw sequencing reads were demultiplexed using CASAVA according to the TruSeq LT index information. The forward read contained 28–30 nt; 18 nt spatial barcode followed by a semi-randomized 9 nt unique molecular identifier (UMI) (RA1–2) or randomized UMI (7 nts, RA3–6), while the reverse read contained the at least 50 nt transcript information. The first five bases in the reverse read were hard trimmed and then the rest of the read was quality trimmed based on the Burrows-Wheeler aligner. Trimmed reads were mapped to the human genome reference (GRCh38) using STAR^48^. Mapped reads were annotated based on Ensembl’s v79 information and then paired with their forward read, UMI-filtered with a Hamming distance of 2 and counted using HTseq-count^49^. Quality control statistics were computed as the number of paired reads per spatial barcode, the number of UMI counts per spatial barcode, and the number of unique gene counts per spatial barcode. Data were normalized per biopsy using a linear regression approach^50^ with a mean gene cut-off per ST spot prior to normalization (minimal size = 200).

#### Image registration, alignment, and visualization

Images were randomly down-sampled to approximately the same image size per patient biopsy. In the RA1 biopsy, all sections were also cropped to contain approximately the same tissue areas. Image background was removed using scikit-image (v0.17.2)^51^ before registering the sections using SCALED_ROTATION (biopsies RA1 and RA2) and RIGID_BODY (biopsies RA3, RA4, RA5, and RA6) from PyStackReg (v0.2.2)^52^. All of the following data processing was performed in R (v.4.0.1). As the spatially resolved data are of the restricted resolution, the data were interpolated using the akima package (v0.6-2.1) over the tissue section area to aid in data visualization.

#### Single-cell segmentation

Single-cell segmentation was performed by combining Ilastik (v1.3.2)^53^ and CellProfiler (v3.1.8)^54^. Random forest classification implemented in Ilastik was used to train three distinct classes (nuclei, membrane, and background) to enable the prediction and export of probability maps. CellProfiler was then used to segment those exported probability maps to create labeled single cell masks for downstream analysis. ST spots with no detected nuclei were removed from further analysis (Supplementary Notes 1 and 2).

#### Coupling single-cell topology to ST data

ST 100 µm barcoded area locations were used to crop areas of 200 × 200 pixels from the corresponding H&E images. These cropped and segmented images were imported into histoCAT^20^ for single-cell quantification and spatial analysis. ST-based phenotypic clusters were matched to the single-cell data as well as the manual infiltrate annotations. Each image was saved as an individual interactive session for histoCAT loading.

#### Phenotyping cell-type calling

We used PhenoGraph^55^ with the code provided at https://github.com/jacoblevine/PhenoGraph to define phenotypic groups (PG) based on the morphological single-cell readouts. We used histoCAT^20^ to extract mean marker expression as well as morphological features from the single-cell mask. The default setting (30 nearest neighbors) was used to define 25 distinct phenotypic groups using a fixed seed for the Louvain method (random seed: 2).

#### Spatially resolved data analysis

To cluster regions, most variable genes were first selected^50^. Briefly, all of the gene counts were first divided by the size factor, i.e., the number of counts in each spot. Then, we computed the mean and coefficient of variation for each gene in all the spatial spots generated per patient sample. Gene with the highest test statistics, i.e., genes with the largest difference between the squared coefficient of variation and the median were selected as those that are the most variable in the data. For patient samples RA1–6, the following numbers of topmost variable genes were chosen respectively: 2000; 2000; 500; 100; 500, and 100. Principal component analysis (PCA) was performed on the subsampled and normalized region × gene expression matrices, followed by two-dimensional *t*-stochastic neighbor embedding (tSNE)^56^. The initial number of PCA dimensions used in tSNE was determined using a permutation test at 5% false discovery rate (FDR). Hierarchical clustering (R stats v4.0.1) was done on the first three tSNE components to determine the number of individual clusters present in the whole tissue volume using the ward.D2 approach followed by differential expression analysis using a likelihood ratio test^57^. For patient samples RA1-6, the following numbers of clusters were predefined in spatial clustering: 4; 3, 3; 4; 4; 4; and the following number of clusters were predefined during the hierarchical clustering of the infiltrate regions: 2; 3, 3, 3, 2, 3. DE genes between the clusters were called as differentially expressed^18^ if satisfying the following criteria: *p* < 0.001 and log-ratio >0.5.

#### Single-cell signatures

Single-cell type signatures were downloaded from Stephenson et al.^14^ and Zhang et al.^15^, and the top 200 markers ${m}_{l}$ were kept for each cell type *l* with the following criteria: average log fold change >1 and FDR < 5%. A total of 16 cell types were present in the references. ST matrix is defined as region × gene matrix for a total of *i* regions and *j* genes. To score each cell type ${c}_{l,r}$ assignment per each individual spatial feature ${S}_{i,j}$, the normalized ST matrix was first subset for ${m}_{l}$ if more than 3 ${m}_{l}$ genes for each ${S}_{i,j}$ were present; creating a *R* × *K* matrix. Then, we computed the correlation coefficient over each ${S}_{i,j}$ for each pair of genes (*j, k*) and a total of *R* regions such that:

1 $$\underline{{X}_{j}}=\frac{1}{R}\mathop{\sum }\limits_{r=1}^{R}{X}_{j,r},\,{{{{{\rm{j}}}}}}=[1,{{{{{\rm{K}}}}}}]$$

2 $${{{{{\mathrm{Cov}}}}}}_{j,k}=\frac{1}{R}\mathop{\sum }\limits_{r=1}^{R}({X}_{j,r}-\underline{{X}_{j}})({X}_{k,r}-\underline{{X}_{k}}),k=[1,K]$$

3 $${{{{{\mathrm{Corr}}}}}}_{j,k}=\frac{{{{{{\mathrm{Cov}}}}}}_{j,k}}{\sqrt{{{{{{\mathrm{Cov}}}}}}_{j,j}{{{{{\mathrm{Cov}}}}}}_{k,k}}}$$

A gene-to-gene co-expression score was considered valid if ${{{{{\mathrm{Corr}}}}}}_{j,k} \, > \, 0$ and these genes *M* were used in all further analysis. Now, the spatial matrix was subset to create a R × M matrix used in the cell typing task and a cell-type expression score ${c}_{l,r}$ for each gene expression value ${Y}_{m,r}$ was calculated:

4 $${c}_{l,r}=\mathop{\sum }\limits_{m=1}^{M}{Y}_{m,r}$$

The cell-type assignment ${c}_{l,r}$ was then scaled between the different cell types present in all the regions:

5 $${c}_{{{{{{\mathrm{max}}}}}}}={{{{{\mathrm{max}}}}}}_{r}{c}_{l,r},$$

6 $${C}_{l,r}=\frac{{c}_{l,r}}{{c}_{{{{{{\mathrm{max}}}}}}}}$$

To represent proportions of cell types in each region, we finally scaled the data by the cumulative cell-type score calculated for the region such that:

7 $${c}_{{{{{{\mathrm{sum}}}}}}}=\mathop{\sum }\limits_{r=1}^{R}{C}_{l,r},\,l=[1,16]$$

8 $${C}_{l,r}=\frac{{C}_{l,r}}{{c}_{{{{{{\mathrm{sum}}}}}}}}$$

${C}_{l,r}$ represented the approximated contribution of each cell type *l* in each region *r*. The gene signatures *M* were also tested for functional enrichment with Gene Ontology terms with PANTHER^58^. We reported all terms at 5% FDR. In order to predict responses between cell types, we mapped our spatially deconvolved data for each spatial region to a CellPhoneDB^59^ receptor-ligand repository. We reported all terms with positive log mean expression ratios >0.1 and *p* < 0.01.

### Reporting summary

Further information on research design is available in the Nature Research Reporting Summary linked to this article.

## Supplementary information

Supplementary Information

Description of Additional Supplementary Files

Supplementary Data 1

Supplementary Data 2

Supplementary Data 3

Reporting Summary

## Data Availability

Raw sequencing data are available at NCBI’s Sequence Read Archive under accession PRJNA794338. All processed data are available at the Single Cell Portal (https://portals.broadinstitute.org/single_cell/study/SCP1414/). Volumetric expression heatmaps can be viewed interactively using an RShiny application (https://spatialtranscriptomics3d.shinyapps.io/3DSeTH/).

## Code availability

All code has been deposited to Zenodo (10.5281/zenodo.5806371).
