# Supplementary material for: Three-dimensional spatial transcriptomics uncovers cell type localizations in the human rheumatoid arthritis synovium
Source: Commun Biol. 2022 Feb 11;5:129. doi: 10.1038/s42003-022-03050-3 (PMC8837632; doi:10.1038/s42003-022-03050-3)
Supplement: Supplementary file 1 — Supplementary Information [file 42003_2022_3050_MOESM1_ESM.pdf]

Supplementary information for:

Three-dimensional spatial transcriptomics uncovers cell type localizations in the human rheumatoid arthritis synovium

Sanja Vickovic[1,2,3,4,*], Denis Schapiro[1,5,6,†], Konstantin Carlberg[7,†], Britta Lötstedt[1,7,†], Ludvig Larsson[7,†], Franziska Hildebrandt[8], Marina Korotkova[9,10], Aase H Hensvold[9,10], Anca I Catrina[9,10], Peter K Sorger[5], Vivianne Malmström[9,10], Aviv Regev[1,11,12], Patrik L Ståhl[7]

†These authors contributed equally to this work

*To whom correspondence should be addressed: vickovic@broadinstitute.org (S.V)

[1]Klarman Cell Observatory, Broad Institute of MIT and Harvard, Cambridge, MA, USA

[2]Department of Biology, Massachusetts Institute of Technology, Cambridge, MA, USA

[3]Science for Life Laboratory, Department of Biochemistry and Biophysics, Stockholm University, Solna, Sweden

[4]New York Genome Center, New York, NY, USA

[5]Laboratory of Systems Pharmacology, Harvard Medical School, Boston, MA, USA

[6]Institute for Computational Biomedicine and Institute of Pathology, Faculty of Medicine, Heidelberg University Hospital and Heidelberg University, Heidelberg, Germany

[7]Science for Life Laboratory, Department of Gene Technology, KTH Royal Institute of Technology, Stockholm, Sweden

[8]Stockholm University, Department of Molecular Biosciences, the Wenner Gren Institute, Stockholm, Sweden

[9]Karolinska Institutet, Division of Rheumatology, Department of Medicine, Center for Molecular Medicine, Stockholm, Sweden

[10]Karolinska University Hospital, Unit of Rheumatology, Stockholm, Sweden

[11]Howard Hughes Medical Institute and Koch Institute for Integrative Cancer Research, Department of Biology, Massachusetts Institute of Technology, Cambridge, MA, USA

[12]Current address: Genentech, 1 DNA Way, South San Francisco, CA, USA

Supplementary Figures

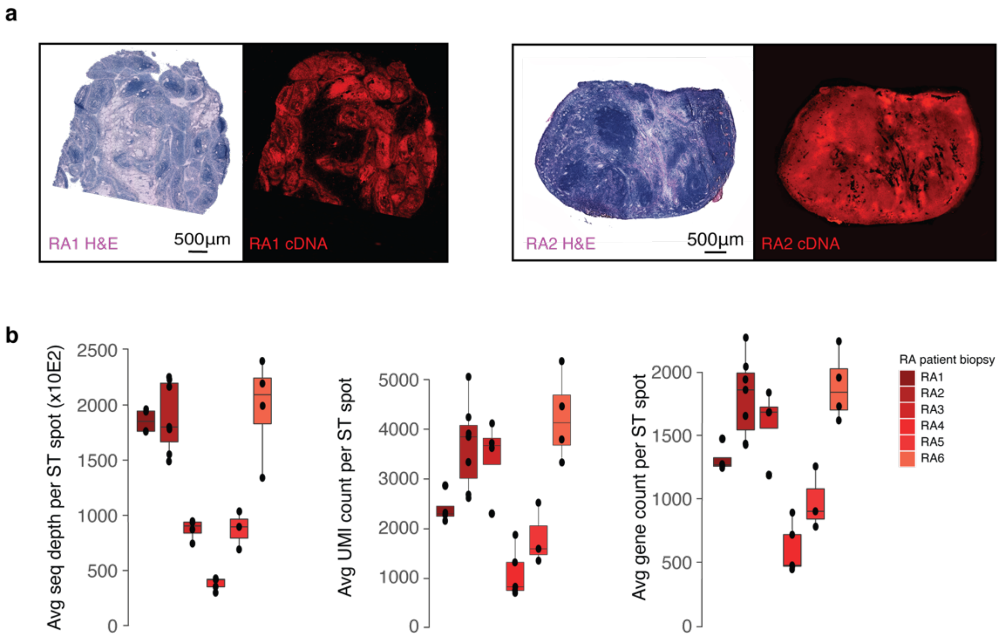

Supplementary Figure 1. Fluorescent footprints and sequencing library statistics. (a) Images of H&E stained tissue sections and corresponding fluorescent cDNA expression footprints marking spatial gene activity for RA1 (knee) and RA2 (hip) patient biopsies. cDNA signal shows optimized tissue handling for both RA sampling sites. Scale bars represent 500µm and are shared between the H&E and cDNA patient images. **(b)** Sequencing library statistics for all patient biopsies (RA1-6; color code) reporting number of raw sequence reads, unique UMIs and unique protein coding genes per 100µm spatial feature (black dots represent average values per section). Center black line, median; black box, interquartile range; black vertical line, 1.5x interquartile range.

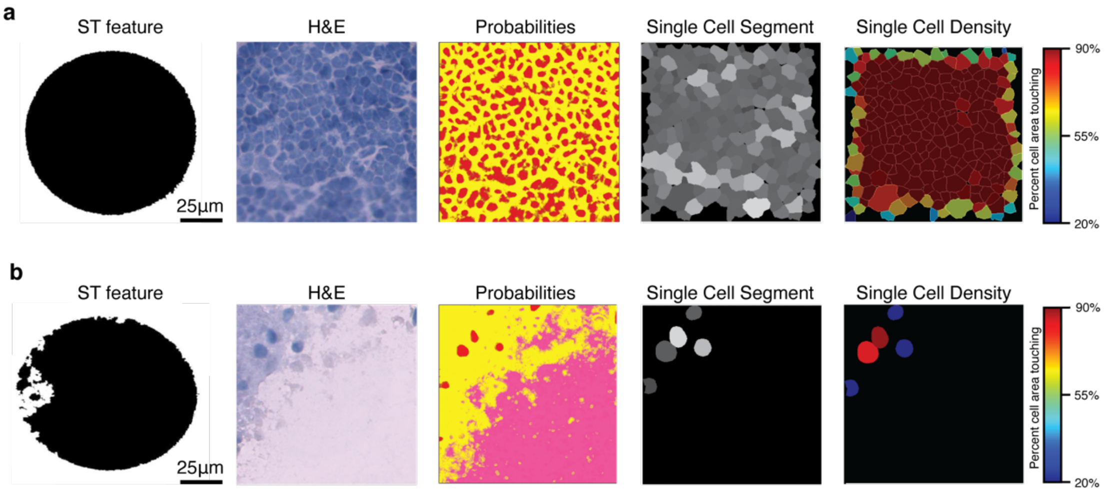

Supplementary Figure 2. Single cell segmentation and histoCAT analysis workflow. (a) 100µm ST features were used to crop the respective H&E images as 100µm x 100µm areas. Ilastik software was used to train a random-forest classifier to create Probabilities for three classes in the H&E image (red: nuclei; yellow: membrane; pink: background). Single Cell Segments (each individual cell represented in discrete grayscale color code) and Single Cell Density (color scale represents the percent of each cell's area touching a membrane of a neighboring cell) were quantified and visualized in the histoCAT software (**Methods**). **(b)** Same as in **(a)** for a less cell dense area overlapping a 100µm ST feature. Scale bars represent 25µm and are shared between all respective images in **(a)** and **(b)**.

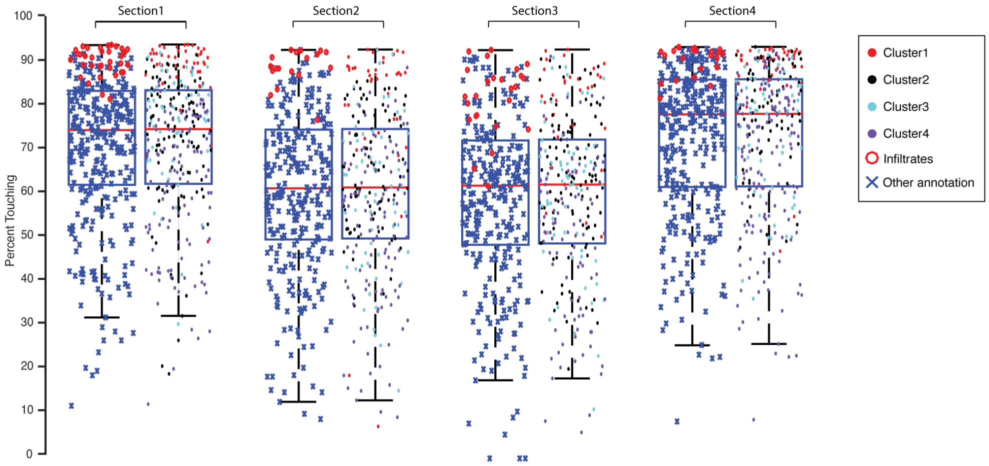

Supplementary Figure 3. Cellular morphology reproduces manual annotations. On average 80% of all manually annotated infiltrates (red octagons) were present in 100µm ST features with a percent touching score >70% (left boxplot in pair), while 91% of all Cluster1 ST features were present in cell dense areas (red circle; right boxplot in pair). Center red line, median; blue box, interquartile range; black dashed vertical line, 1.5x interquartile range.

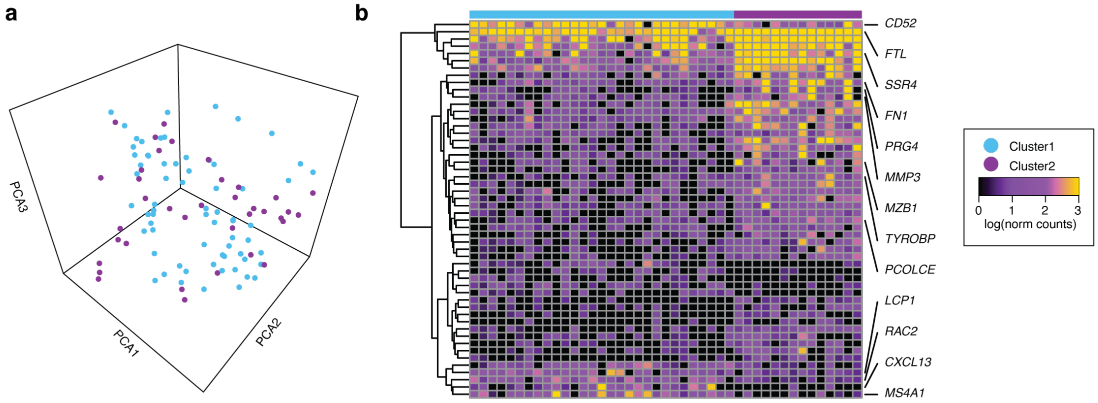

Supplementary Figure 4. Clustering analysis of RA1 infiltrate regions. (a) PCA plot of each individual spatially resolved infiltrate feature present in any of the RA1 tissue sections. Samples have been color-coded based on hierarchical clusters (cyan; purple). **(b)** Heatmap of differentially expressed genes (color scale, rows) between the two clusters (color code, columns) as determined in **(a)**. Color code is shared between the panels.

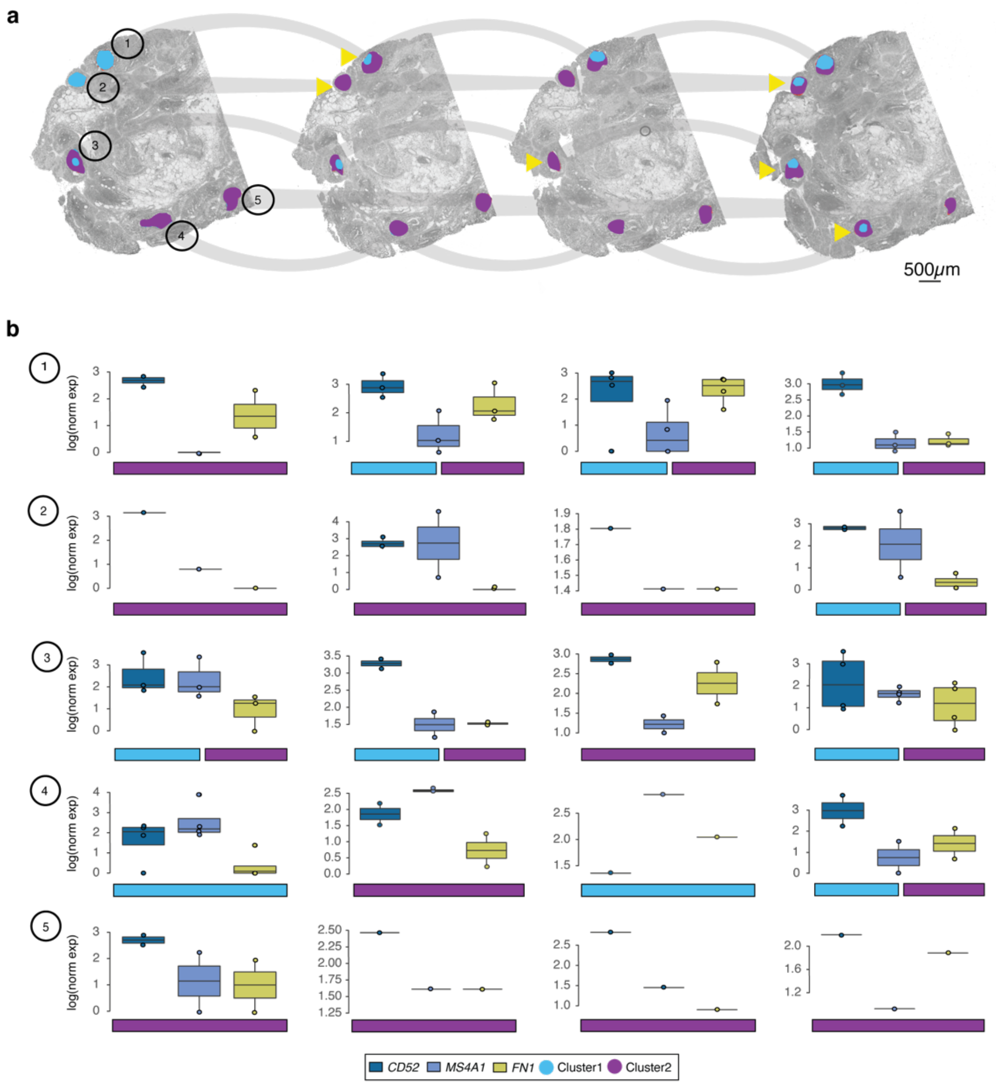

Supplementary Figure 5. Average infiltrate signatures in four RA1 biopsy sections. (a) Volumetric morphological view with overlaid color-coded infiltrate regions present in all four sections as determined by clustering (color code). Yellow arrow marks an event where the infiltrate region changed its cluster assignment in the 3D tissue volume. Scale bar represents 500µm and is shared between the sections. **(b)** Boxplots showing average expression of three cluster driving genes; *CD52*; *MS4A1* and *FN1* (color code; blue; light blue; yellow) present in two spatial infiltrate clusters (color bar; cyan; purple). Center black line, median; color-coded box, interquartile range; black vertical line, 1.5x interquartile range. In case only one spatial feature was present, only individual measurements were plotted (dots). Infiltrate regions are number coded (1-5) and the numbering is shared between the panels.

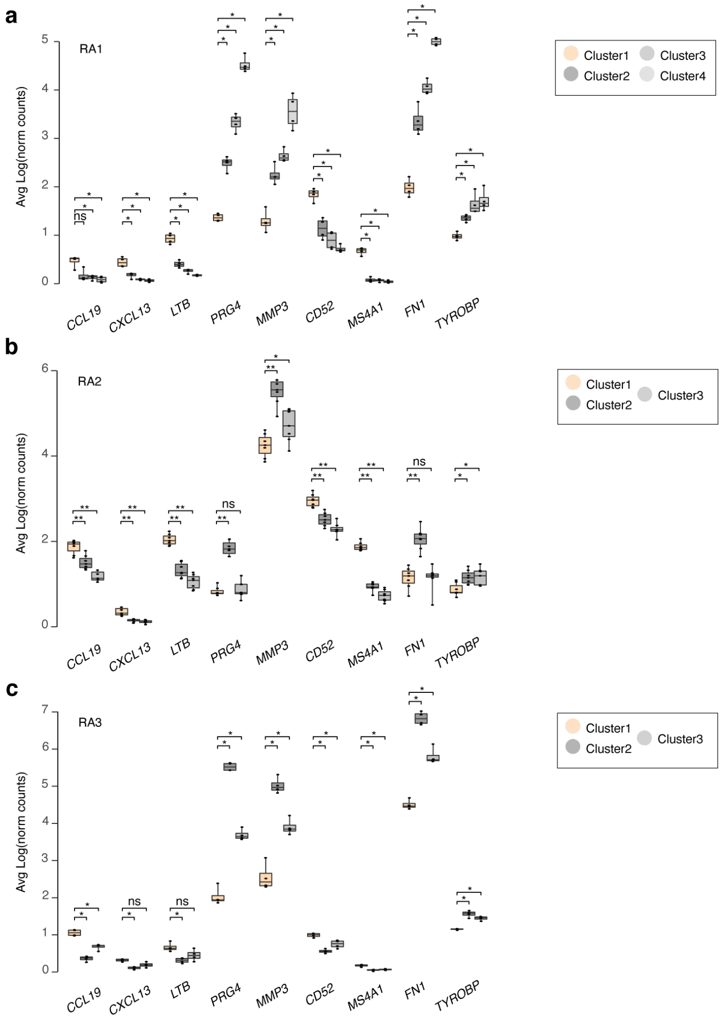

Supplementary Figure 6. Average expression of spatially variable genes in seropositive RA tissue volumes and clusters. (a-c) Boxplots denoting average gene expression (columns) per spatial cluster (color code) in respective seropositive RA tissue volumes (RA1-3). Statistical significance markings (two-sided *t*-test, Benjamini-Hochberg adjusted) are displayed; p>0.05 (ns), 0.005<p≤0.05(*), 0.0005<p≤0.005(**). Center black line, median; color-coded box, interquartile range; black vertical line, 1.58x interquartile range; black dots; mean gene expression values per individual tissue section.

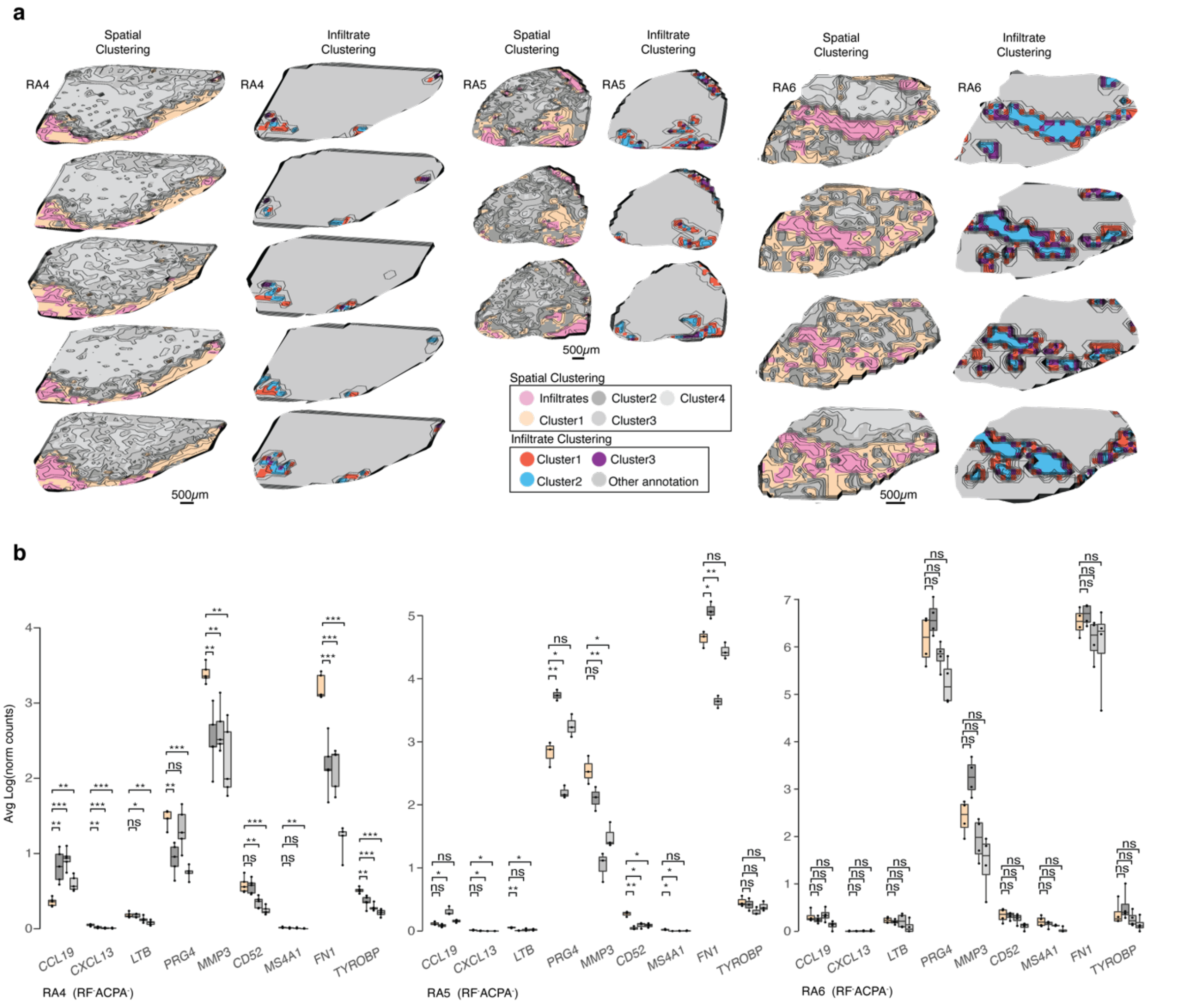

Supplementary Figure 7. Spatial and infiltrate clustering for seronegative patient biopsies. **(a)** Spatial and infiltrate clustering (color code). Scale bars represent 500µm and are shared between the sections within the individual patient tissue volume. **(b)** Boxplots denoting average expression (columns) per spatial cluster (color code) in respective seronegative RA tissue volumes (RA4-6). Statistical significance markings (two-sided *t*-test, Benjamini-Hochberg adjusted) are displayed; p>0.05 (ns), 0.005<p≤0.05(*), 0.0005<p≤0.005(**), p≤0.0005(***). Center black line, median; color-coded box, interquartile range; black vertical line, 1.58x interquartile range; black dots; mean gene expression values per individual tissue section. Spatial clustering color code is shared between panels **(a)** and **(b)**.

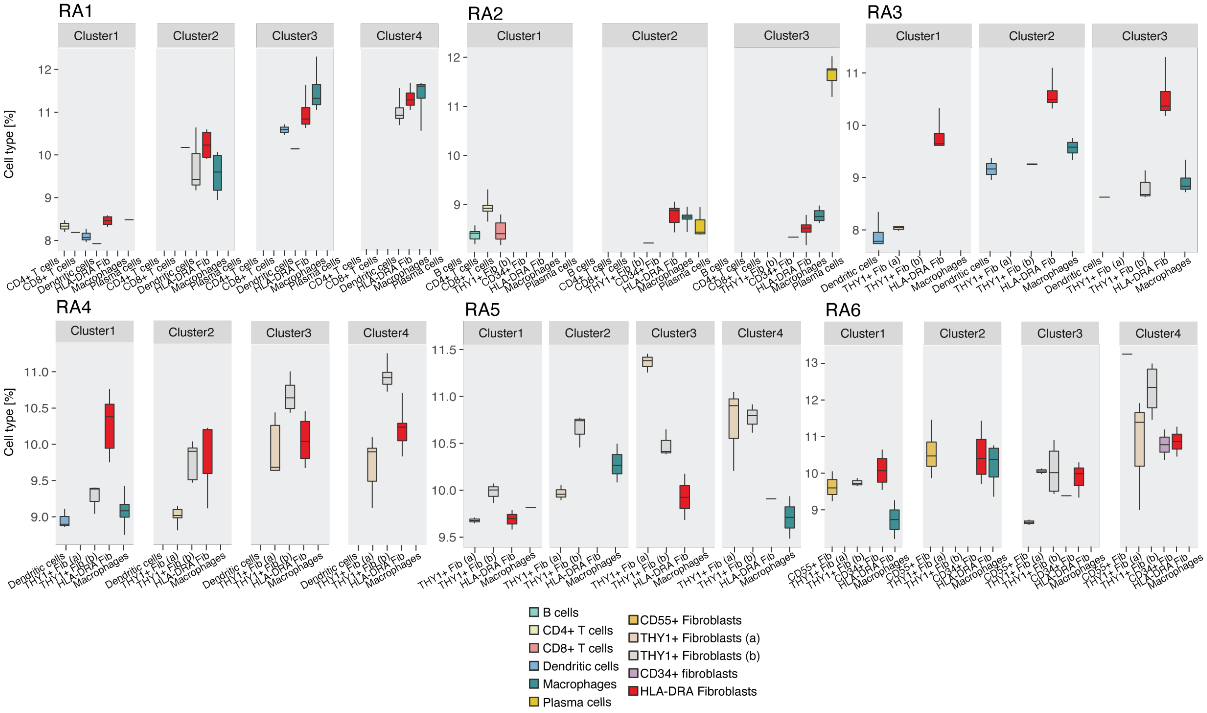

Supplementary Figure 8. Cell type abundances across spatial clusters. Boxplots denoting top three cell type abundances [%] (columns; color code) in each spatial cluster in respective seropositive (RA1-3) and seronegative (RA4-6) tissue volumes. Center black line, median; color-coded box, interquartile range; black vertical line, 1.58x interquartile range.

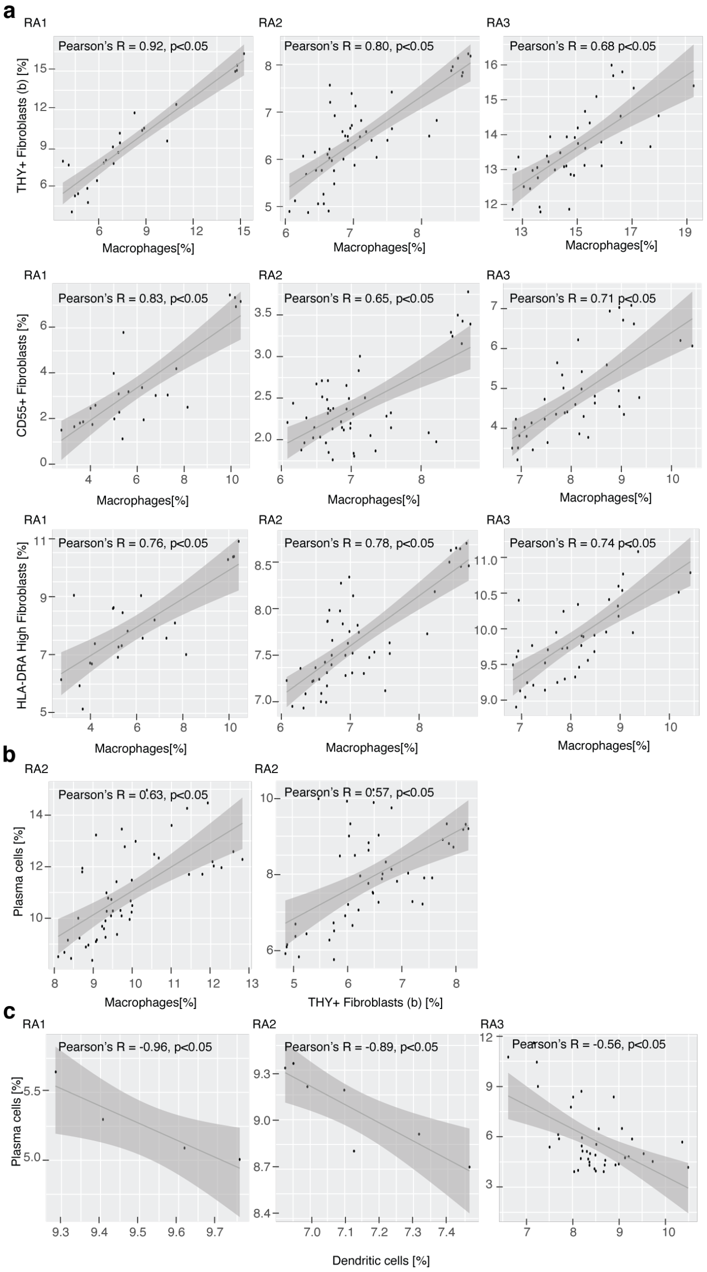

Supplementary Figure 9. Correlations between spatial cell type abundances in the rheumatoid arthritis synovium. (a) Correlation plots between macrophages and different fibroblast subtype abundances in the whole tissue volume in seropositive patient samples (RA1-3). **(b)** Correlation plots between macrophage, fibroblast and plasma cell abundances in RA2. **(c)** Correlation plots between regional distributions of macrophage and dendritic cell abundances in seropositive RA tissue volumes. Reported are Pearson's correlation coefficients (R) and p values for each comparison. Colored line (gray) represents the linear regression line while the shaded areas represent the 95% confidence intervals for predictions from the linear model.

Supplementary Tables

Sample	Rheumatoid factor	ACPA status	Sex	Age	Biopsy site	Disease duration at time-point	Treatment at sampling
RA1	RF-positive	positive	F	70-74 years old	knee	Approx. 18 years	MTX
RA2	RF-positive	positive	F	50-54 years old	hip	Approx. 14 years	MTX and anti-TNF
RA3	RF-positive	positive	F	80-84 years old	knee	Approx. 17 years	MTX
RA4	RF-negative	negative	M	35-39 years old	hip	Approx. 20 years	MTX and anti-IL6R
RA5	RF-negative	negative	M	40-44 years old	knee	Approx. 23 years	MTX and anti-IL6R
RA6	RF-negative	negative	F	55-59 years old	hip	Approx. 6 years	MTX and CTLA4-Ig

Supplementary Table 1. RA patient information.

Supplementary Notes

Supplementary Note 1. In RA1.3 location 24_3 does not have any cells in the H&E image.

Supplementary Note 2. In RA1.3 location 30_17 is missing and was so far removed from Cluster 2.
