# Supplementary material for: Three-dimensional spatial transcriptomics uncovers cell type localizations in the human rheumatoid arthritis synovium
Source: Commun Biol. 2022 Feb 11;5:129. doi: 10.1038/s42003-022-03050-3 (PMC8837632; doi:10.1038/s42003-022-03050-3)
Supplement: Supplementary file 2 — Description of Additional Supplementary Files [file 42003_2022_3050_MOESM2_ESM.pdf]

Description of Additional Supplementary Files

File name: Supplementary Data 1

Description: Differentially expressed genes in infiltrate structures (RA1-6).

File name: Supplementary Data 2

Description: Differentially expressed genes in all tissue sections (RA1-6).

File name: Supplementary Data 3

Description: Spatial cell type signatures in all tissue sections (RA1-6).
