# Supplementary material for: Three-dimensional spatial transcriptomics uncovers cell type localizations in the human rheumatoid arthritis synovium
Source: Commun Biol. 2022 Feb 11;5:129. doi: 10.1038/s42003-022-03050-3 (PMC8837632; doi:10.1038/s42003-022-03050-3)
Supplement: Supplementary file 6 — Reporting Summary [file 42003_2022_3050_MOESM6_ESM.pdf]

nature research

Corresponding author(s): Sanja Vickovic

Last updated by author(s): November 17, 2021

Reporting Summary

Nature Research wishes to improve the reproducibility of the work that we publish. This form provides structure for consistency and transparency in reporting. For further information on Nature Research policies, see our Editorial Policies and the Editorial Policy Checklist.

Statistics

For all statistical analyses, confirm that the following items are present in the figure legend, table legend, main text, or Methods section.

n/a	Confirmed	
☐	☒	The exact sample size (*n*) for each experimental group/condition, given as a discrete number and unit of measurement
☐	☒	A statement on whether measurements were taken from distinct samples or whether the same sample was measured repeatedly
☐	☒	The statistical test(s) used AND whether they are one- or two-sided<br>*Only common tests should be described solely by name; describe more complex techniques in the Methods section.*
☐	☒	A description of all covariates tested
☐	☒	A description of any assumptions or corrections, such as tests of normality and adjustment for multiple comparisons
☐	☒	A full description of the statistical parameters including central tendency (e.g. means) or other basic estimates (e.g. regression coefficient) AND variation (e.g. standard deviation) or associated estimates of uncertainty (e.g. confidence intervals)
☐	☒	For null hypothesis testing, the test statistic (e.g. *F*, *t*, *r*) with confidence intervals, effect sizes, degrees of freedom and *P* value noted<br>*Give P values as exact values whenever suitable.*
☒	☐	For Bayesian analysis, information on the choice of priors and Markov chain Monte Carlo settings
☒	☐	For hierarchical and complex designs, identification of the appropriate level for tests and full reporting of outcomes
☐	☒	Estimates of effect sizes (e.g. Cohen's *d*, Pearson's *r*), indicating how they were calculated

Our web collection on statistics for biologists contains articles on many of the points above.

Software and code

Policy information about availability of computer code

Data collection	Data was pre-processed using a recently published ST pipeline. Raw sequencing reads were demultiplexed using CASAVA according to the TruSeq LT index information. The forward read contained 28-30 nt; 18 nt spatial barcode followed by a semi-randomized 9 nt unique molecular identifier (UMI) (RA1-2) or randomized UMI (7 nts, RA3-5), while the reverse read contained the 50 nt transcript information. The first five bases in the reverse read were hard trimmed and then the rest of the read was quality trimmed based on the Burrows-Wheeler aligner. Trimmed reads were mapped to the human genome reference (GRCh38) using STAR40. Mapped reads were annotated based on Ensembl's v79 information and then paired with their forward read, UMI-filtered with a Hamming distance of 2 and counted using HTseq-count. Quality control statistics were computed as number of paired reads per spatial barcode; number of UMI counts per spatial barcode and number of unique gene counts per spatial barcode. Images were randomly down-sampled to approximately the same image size per patient biopsy.
Data analysis	In the RA1 biopsy, all sections were also cropped to contain approximately the same tissue areas. Image background was removed using scikit-image43 before registering the sections using SCALED_ROTATION (biopsies RA1 and RA2) and RIGID_BODY (biopsies RA3, RA4, RA5, RA6) from PyStackReg. Single cell segmentation was performed by combining Ilastik 1.3.2 and CellProfiler 3.1.8. Random forest classification implemented in Ilastik was used to train three distinct classes (nuclei, membrane, and background) to enable the prediction and export of probability maps. CellProfiler was then used to segment those exported probability maps to create labeled single cell masks for downstream analysis. We used PhenoGraph with the code in https://github.com/jacoblevine/PhenoGraph to define phenotypic groups (PG) based on the morphological single cell readouts. We used histoCAT to extract mean marker expression as well as morphological features from the single cell mask. The default setting (30 nearest neighbors) was used to define 25 distinct phenotypic groups using a fixed seed for the Louvain method (random seed: 2). All code has been deposited to https://github.com/klarman-cell-observatory/3dst.

For manuscripts utilizing custom algorithms or software that are central to the research but not yet described in published literature, software must be made available to editors and reviewers. We strongly encourage code deposition in a community repository (e.g. GitHub). See the Nature Research guidelines for submitting code & software for further information.

Data

Policy information about availability of data

All manuscripts must include a data availability statement. This statement should provide the following information, where applicable:

- Accession codes, unique identifiers, or web links for publicly available datasets
- A list of figures that have associated raw data
- A description of any restrictions on data availability

Raw sequencing data is available through an MTA with Vivianne Malmström (vivianne.malmstrom@ki.se). All processed data files are available at the Single Cell Portal (https://portals.broadinstitute.org/single_cell/study/SCP460/).

Field-specific reporting

Please select the one below that is the best fit for your research. If you are not sure, read the appropriate sections before making your selection.

- [x] Life sciences
- [ ] Behavioural & social sciences
- [ ] Ecological, evolutionary & environmental sciences

For a reference copy of the document with all sections, see nature.com/documents/nr-reporting-summary-flat.pdf

Life sciences study design

All studies must disclose on these points even when the disclosure is negative.

Sample size	Study contained two patient groups: seropositive and seronegative rheumatoid arthritis. Each patient group contained a minimum of three replicate tissues per category and each tissue was processed in at least 3 replicates. Spatial power analysis indicates the study using all the spatial points is at least 70% powered.
Data exclusions	No data was excluded from the analyses.
Replication	A total of min n=3 replicates (ie. tissue sections) per human specimen were analyzed.
Randomization	NA
Blinding	Blinding was not relevant for this study.

Reporting for specific materials, systems and methods

We require information from authors about some types of materials, experimental systems and methods used in many studies. Here, indicate whether each material, system or method listed is relevant to your study. If you are not sure if a list item applies to your research, read the appropriate section before selecting a response.

Materials & experimental systems

n/a	Involved in the study	
x		Antibodies
x		Eukaryotic cell lines
x		Palaeontology and archaeology
x		Animals and other organisms
	x	Human research participants
x		Clinical data
x		Dual use research of concern

Methods

n/a	Involved in the study	
x		ChIP-seq
x		Flow cytometry
x		MRI-based neuroimaging

Human research participants

Policy information about studies involving human research participants

Population characteristics	Synovial tissue biopsies from knee or hip joints were obtained during orthopedic replacement surgery. Additional patient information can be found in Supplementary Table 1. All patients in the study were exhibited long lasting disease with RF (rheumatoid factor) positive clinical associations.
Recruitment	RF (rheumatoid factor) positive patients. Two subsets: seropositive and seronegative.
Ethics oversight	Ethical approvals were granted by the Ethics Committee of Karolinska University Hospital (2009/1262-31/3) and patients gave their informed written consent to participate in the study.

Note that full information on the approval of the study protocol must also be provided in the manuscript.
